# Modulating sphingosine 1-phosphate receptor signaling skews intrahepatic leukocytes and attenuates murine nonalcoholic steatohepatitis

**DOI:** 10.3389/fimmu.2023.1130184

**Published:** 2023-04-21

**Authors:** Chieh-Yu Liao, Fanta Barrow, Nanditha Venkatesan, Yasuhiko Nakao, Amy S. Mauer, Gavin Fredrickson, Myeong Jun Song, Tejasav S. Sehrawat, Debanjali Dasgupta, Rondell P. Graham, Xavier S. Revelo, Harmeet Malhi

**Affiliations:** ^1^ Division of Gastroenterology and Hepatology, Mayo Clinic, Rochester, MN, United States; ^2^ Department of Integrative Biology and Physiology, University of Minnesota, Minneapolis, MN, United States; ^3^ Department of Internal Medicine, College of Medicine, The Catholic University of Korea, Seoul, Republic of Korea; ^4^ Division of Anatomic Pathology, Department of Laboratory Medicine and Pathology, Mayo Clinic, Rochester, MN, United States

**Keywords:** lipotoxicity, fatty liver, sphingolipids, Etrasimod, Amiselimod

## Abstract

Sphingosine 1-phosphate (S1P) is a bioactive sphingolipid associated with nonalcoholic steatohepatitis (NASH). Immune cell-driven inflammation is a key determinant of NASH progression. Macrophages, monocytes, NK cells, T cells, NKT cells, and B cells variably express S1P receptors from a repertoire of 5 receptors termed S1P_1_ – S1P_5_. We have previously demonstrated that non-specific S1P receptor antagonism ameliorates NASH and attenuates hepatic macrophage accumulation. However, the effect of S1P receptor antagonism on additional immune cell populations in NASH remains unknown. We hypothesized that S1P receptor specific modulation may ameliorate NASH by altering leukocyte recruitment. A murine NASH model was established by dietary feeding of C57BL/6 male mice with a diet high in fructose, saturated fat, and cholesterol (FFC) for 24 weeks. In the last 4 weeks of dietary feeding, the mice received the S1P_1,4,5_ modulator Etrasimod or the S1P_1_ modulator Amiselimod, daily by oral gavage. Liver injury and inflammation were determined by histological and gene expression analyses. Intrahepatic leukocyte populations were analyzed by flow cytometry, immunohistochemistry, and mRNA expression. Alanine aminotransferase, a sensitive circulating marker for liver injury, was reduced in response to Etrasimod and Amiselimod treatment. Liver histology showed a reduction in inflammatory foci in Etrasimod-treated mice. Etrasimod treatment substantially altered the intrahepatic leukocyte populations through a reduction in the frequency of T cells, B cells, and NKT cells and a proportional increase in CD11b^+^ myeloid cells, polymorphonuclear cells, and double negative T cells in FFC-fed and control standard chow diet (CD)-fed mice. In contrast, FFC-fed Amiselimod-treated mice showed no changes in the frequencies of intrahepatic leukocytes. Consistent with the improvement in liver injury and inflammation, hepatic macrophage accumulation and the gene expression of proinflammatory markers such as *Lgals3* and *Mcp-1* were decreased in Etrasimod-treated FFC-fed mice. Etrasimod treated mouse livers demonstrated an increase in non-inflammatory (*Marco*) and lipid associated (*Trem2*) macrophage markers. Thus, S1P_1,4,5_ modulation by Etrasimod is more effective than S1P_1_ antagonism by Amiselimod, at the dose tested, in ameliorating NASH, likely due to the alteration of leukocyte trafficking and recruitment. Etrasimod treatment results in a substantial attenuation of liver injury and inflammation in murine NASH.

## 1 Introduction

The most common chronic liver disease in the United States, nonalcoholic fatty liver disease (NAFLD) is a heterogenous group of disorders whose spectrum ranges from isolated steatosis to nonalcoholic steatohepatitis (NASH) (1). Lipid accumulation is the hallmark of isolated steatosis, whereas NASH is a lipotoxic disorder histologically characterized by steatosis, lobular inflammation and hepatocellular ballooning, which all together lead to increased fibrosis risk (2). The histological prominence of steatosis coupled with lipidomic abnormalities noted in NASH and mechanistic studies in lipotoxic model systems have suggested a role for bioactive lipids in NASH pathogenesis, including sphingolipids (3, 4). These bioactive lipids can be generated due to activation of the hepatocellular endoplasmic reticulum stress response leading to *de novo* sphingolipid synthesis and ensuing release of sphingosine 1-phosphate (S1P) enriched proinflammatory extracellular vesicles (3). We have shown that S1P-enriched extracellular vesicles mediate the recruitment of proinflammatory monocyte-derived macrophages into the liver in NASH (5). However, several immune cell subsets are perturbed during NASH progression and the broader effects of inhibition of S1P signaling on such additional immune cells remain unknown.

S1P is formed from the hydrolysis of ceramide to generate sphingosine which is then phosphorylated by either sphingosine kinase 1 or 2 to form S1P (6). S1P binds five subtypes of G-protein couple receptors (GPCRs), namely S1P_1_ – S1P_5_. S1P receptors transduce intracellular signals to mediate diverse cellular responses including migration, differentiation, proliferation, lymphocyte circulation, as well as T and B cell trafficking (7–9). S1P receptors are widely expressed across various immune cell types (7). In our previous study using a dietary NASH mouse model, we found that treatment with FTY720, an S1P_1, 3, 4, 5_ functional antagonist, ameliorates the cardinal features of NASH (10). Here we utilized Etrasimod, a fully functional antagonist of mouse and human S1P_1_ and partial S1P_4_ and S1P_5_ antagonist (11), and Amiselimod, a functional S1P_1_ antagonist (12), to examine the effects of S1P receptor pharmacological inhibition on the intrahepatic leukocyte populations associated with NASH. Additionally, we compared the effects of Etrasimod to Amiselimod to determine if S1P_1_ receptor antagonism was sufficient to attenuate NASH (13). We report that selective modulation of S1P receptors 1, 4, and 5 through Etrasimod is more effective than S1P_1_ modulation by Amiselimod in improving NASH in a mouse model. Etrasimod reduces the accumulation of macrophages in the liver and consequently reduces the infiltration of inflammatory cells including T cells, B cells and NKT cells, leading to attenuation of liver injury and inflammation.

## 2 Materials and methods

### 2.1 Mouse studies

Animal use was approved by the institutional care and animal use committee (IACUC) of the Mayo Clinic and conducted in accordance with the public health policy on the humane use and care of laboratory animals. C57BL/6J male mice were purchased from Jackson laboratory (Bar Harbor, Maine) at 6 weeks of age. When 12 weeks old, mice were randomized to receive either a diet high in saturated fat, fructose, and cholesterol (FFC, AIN-76A Western Diet, Test Diets 5342) or standard rodent chow diet (CD) for 24 weeks. As previously described by us, at 24 weeks of feeding, this diet recapitulates cardinal features of human NASH including histologic parameters of steatosis, inflammation, ballooning, fibrosis and metabolic parameters of obesity and insulin resistance (14). Mice had unrestricted access to food and water, and were housed in standard pathogen-free facilities, with 12-hour day-night circadian cycles. Twenty weeks into the feeding study mice started receiving drug or vehicle by daily oral gavage for 1 month. The drugs employed were Etrasimod (Cayman Chemical, 21661) or Amiselimod hydrochloride (Cayman Chemical, 20970). Etrasimod was dissolved at 20 mg/mL in DMSO and then diluted to 1.44 mg/mL in 0.5% sodium methylcellulose and administered to mice at a dose of 3 mg/Kg. Amiselimod was dissolved in DMSO at a concentration of 20 mg/mL and then diluted to 1 mg/mL in 0.5% sodium methylcellulose and administered to mice at a dose of 2 mg/Kg. A matched volume of DMSO, similarly diluted in 0.5% sodium methylcellulose, served as the vehicle. Each treatment group (Etrasimod or Amiselimod) had its own corresponding vehicle cohort. Upon completion of the study, liver and plasma were collected after euthanasia. Prior to liver excision, 10 mL PBS was flushed through the liver via inferior vena cava (IVC) cannulation in a retrograde fashion. Then the liver was excised, weighed, and divided for downstream analyses. Approximately 1g of liver tissue per mouse was processed for flow cytometry, described below, and the rest divided for several assays including: cryopreservation (snap frozen in liquid nitrogen) for RNA extraction and protein extraction, fixation in 10% neutral buffered formalin for paraffin embedding, and cryo-sectioning for histological analysis. Platelet poor plasma was isolated from citrated blood (described below) and stored at -20°CC till further analyses. Archived flash frozen, in liquid nitrogen, liver tissue of C57BL/6J wild type mice fed chow or FFC diet for 24 weeks were employed for RNA isolation and quantification of whole liver S1P_1-5_ expression (15).

### 2.2 Cell culture studies

Bone marrow-derived macrophages (BMDM) were isolated from the hind legs of untreated wild type C57BL/6 mice as previously described by us (10). BMDM media was changed every 2 days and on day 7 differentiated macrophages were dissociated with Accutase (Invitrogen, 00-4555-56) for use in experiments. After dissociation, BMDMs were plated in two 100 mm petri dishes and were allowed to attach for 16 hours. Next, BMDMs were serum starved for 2 hours after which they were treated with palmitate (400 µM) complexed to 1% bovine serum albumin and volume-matched vehicle (isopropanol) for 8 hours as described by us (16). Cells were lysed in TRIzol reagent (Invitrogen, 15596026) and lysates were stored at -80°C for RNA extraction. Primary mouse hepatocytes (PMH) were isolated based on standardized techniques as described previously (17). PMHs were treated with palmitate (400 µM) and volume-matched vehicle (isopropanol) for 8 hours and collected in TRIzol reagent and stored at -80°C for RNA extraction.

### 2.3 Glucose tolerance testing

Intraperitoneal glucose tolerance test (GTT) was performed on mice in both CD and FFC-fed cohorts at 22 weeks on diet. Mice were fasted overnight for 16 hours and injected with 20% glucose intraperitoneally at a dose of 2 mg/Kg body mass. Blood glucose values were acquired before injection and 15, 30, 45, 60, 90, 120, and 150 minutes after injection. Tail vein blood sampling was done using AssurePlatinum blood glucose meter and test strips (Arkray, 67841996). The area under the glucose tolerance curve was calculated in Microsoft Excel and glucose disposal capacity was compared between groups.

### 2.4 Histology and immunohistochemistry

Five μm sections of formalin-fixed, paraffin-embedded (FFPE) liver tissues were stained with hematoxylin and eosin (H&E) using standard techniques at the Mayo Clinic Histology Core. Histology was observed and H&E slides were graded for steatosis and inflammation using the NAFLD activity score. Immunohistochemistry for Mac-2/Galectin-3 (Invitrogen, 14-5301-85) and Ki67 (Abcam, ab16667) was performed to assess macrophage accumulation and hepatocyte proliferation respectively. Five μm FFPE liver sections were dewaxed with xylene and rehydrated through graded 100%, 95%, 70%, and 50% ethanol sequentially. Slides were then immersed in 10 mM sodium citrate buffer with 0.05% Tween-20 (pH 6.0) for 20 minutes at 95°C for epitope retrieval. The slides were allowed to cool on the benchtop to room temperature. After cooling, endogenous peroxidase activity was blocked by incubating the slides in 3% hydrogen peroxide for 10 minutes. For Mac-2 staining, non-specific immunoglobulin binding was blocked using the Rat-IgG ABC Kit (Vector Laboratories, PK 4004) time for 30 minutes, followed by the Rodent Block M solution (Biocare Medical, RBM 961). For Ki67 blocking was performed using Rabbit-IgG ABC Kit (Vector Laboratories, PK 4001) for 30 minutes. All slides were further blocked with avidin and biotin blocking solution for 15 minutes each using the Avidin/Biotin Blocking Kit (Vector Laboratories, SP 2001). Primary antibodies for Mac-2 (1:200 dilution) and Ki67 (1:200 dilution) were applied and incubated overnight at 4°C in humidified chambers. Slides were washed and incubated for 30 minutes each in biotinylated secondary antibody (1:500 dilution) and ABC reagent from the ABC Kit per manufacturer’s instructions. Signals were visualized using the ImmPACT DAB Peroxidase Substrate Kit (Vector Laboratories, SK 4105), and counterstained with Nuclear Fast Red Solution for Mac-2 (MilliporeSigma, N 8002) for 20 minutes and with Hematoxylin for Ki67 for 30 seconds. Sections were dehydrated through graded ethanol and mounted using Organo/Limonene Mount (Santa Cruz, SC-45087). The positive areas were quantified using the NIS-Elements imaging software (Nikon) on a Nikon microscope with a DS-U3 camera (Nikon, Japan) on the 20x objective lens. Images with preserved settings for light and exposure were used during image analysis and area quantification for Mac-2. Ten high power fields (20x objective lens) were manually counted for nuclear positivity for quantification of Ki67 signal and expressed as average positive nuclei per high power field. Immunohistochemistry for hepatocyte nuclear factor 4 alpha (HNF4α) (Abcam, ab201460) was performed to assess hepatic differentiation as above except for antigen retrieval, which was performed by immersing slides in Tris-EDTA Buffer with 0.05% Tween-20 (pH 9.0) for 30 minutes at 95°C. Non-specific immunoglobulin binding was blocked using the Rabbit-IgG ABC Kit for 1 hour. Primary antibody for HNF4α (1:250 dilution) was applied and incubated overnight at 4°C in humidified chambers. Representative images were captures with the 10x objective lens.

### 2.5 Flow cytometry

Mouse intrahepatic leukocytes were isolated according to the published method of Blom et al. (18). Following euthanasia mouse livers were perfused with 10 mL PBS in a retrograde manner via IVC. The perfused livers were dissected out of the abdomen and the gall bladder was removed. Next, the livers were disrupted using a gentleMACS tissue dissociator in 5 mL RPMI-1640 per manufacturer’s recommended preset program for 45 seconds. The dissociate was transferred to a 50 mL conical tube and RPMI-1640 was added to a volume of 40 mL. This suspension was centrifuged at 60 x g for 1 minute at room temperature and the supernatant containing hepatic immune cells was collected and centrifuged again at 480 x g for 8 minutes at room temperature. The pellet was resuspended in 10 mL of 37.5% Percoll (MilliporeSigma, P1644-1L) and centrifuged at 850 x g for 30 minutes at room temperature with no brake. The supernatant was aspirated, and the pellet was resuspended in 5 mL of red blood cell lysis buffer for 5 minutes on ice. Lysis buffer was neutralized by adding 10 mL PBS, and the remaining cells were pelleted down by centrifugation. The cells were resuspended in 200 μL of PBS and counted using the Muse® Cell Analyzer (MilliporeSigma). Approximately 1 million cells were transferred to FACS tubes and stained with 1:200 zombie aqua (Biolegend) to discriminate between viable and dead cells. Cells were washed with cell staining buffer (Biolegend) before blocking non-specific binding with TruStain FcX Plus (Biolegend). Staining for cell surface markers was performed with fluorophore-conjugated primary antibodies; CD45.2 (AF700, clone 104, Biolegend), CD3 (BUV737, clone 145-2C11, BD Bioscience), NK1.1 (APC, clone PK136, Biolegend), CD4 (BUV737, clone RM4-5, BD Bioscience), CD8a (BUV805, clone 53-6.7, BD Bioscience), B220 (BV421, clone RA3-6B2, Biolegend), CD19 (BV785, clone 6D5, Biolegend), CD11c (BV711, clone N418, Biolegend), CD11b (PE/Cy7, clone M1/70, Biolegend), and Ly6G (PE, clone 1A8, Biolegend) for 30 minutes at 4°C. Flow cytometry data was acquired on BD Fortessa X-20 and Fortessa X-30 H0081 (BD Bioscience) cytometers and analyzed using FlowJo™ Software version 10.6.1 (Becton, Dickinson & Company).

### 2.6 RNA purification and quantitative real time PCR

Cryopreserved mouse liver tissues of vehicle and treatment groups for both diets were recovered and homogenized in TRIzol reagent. Tissue debris was centrifuged down and the supernatant was transferred into separate Eppendorf tubes. Total RNA was extracted using the Direct-zol RNA Zymo Plus Mini Prep (Zymo Research, R 2070), then the RNA yield and quality was assessed using a NanoDrop ND1000 (ThermoScientific, Waltham, MA). 1 μg of total RNA was reverse transcribed into cDNA with the iScript cDNA Synthesis Kit (Bio-Rad Laboratories, 1708891). Gene expression was analyzed using quantitative real time PCR (qPCR) with gene specific mouse primers (**Table 1**). qPCR was performed using the LightCycler 480 SYBR Green I Master Mix (Roche Diagnostics, 04707516001)), and run on LightCycler 480 (Roche). Each dataset of the target gene of interest was normalized to the geometric mean of the reference genes Hprt and 18S. All datasets from drug treated mice are expressed as fold change relative to vehicle controls in the CD group. For isolated cells, the data represent fold change compared to vehicle controls.

**Table 1:** Mouse qPCR Primers.

Gene	Forward Primer	Reverse Primer
18S	CGCTTCCTTACCTGGTTGAT	GAGCGACCAAAGGAACCATA
Hprt	TCAGTCAACGGGGGACATAAA	GGGGCTGTACTGCTTAACCAG
Cd68	TGTCTGATCTTGCTAGGACCG	GAGAGTAACGGCCTTTTTGTGA
Lgals3	TGGGCACAGTGAAACCCAAC	TCCTGCTTCGTGTTACACACA
Ly6c	GCAGTGCTACGAGTGCTATGG	ACTGACGGGTCTTTAGTTTCCTT
Tnfa	CCCTCACACTCAGATCATCTTCT	GCTACGACGTGGGCTACAG
Il1b	GCAACTGTTCCTGAACTCAACT	ATCTTTTGGGGTCCGTCAACT
Timd4	AGAATGTGCGCTTGGAGCTGAG	GGTTGGGAGAACAGATGTGGTC
Marco	ACAGAGCCGATTTTGACCAAG	CAGCAGTGCAGTACCTGCC
Trem2	CTGGAACCGTCACCATCACTC	CGAAACTCGATGACTCCTCGG
Mcp1	TTAAAAACCTGGATCGGAACCAA	GCATTAGCTTCAGATTTACGGGT
S1pr1	ATGGTGTCCACTAGCATCCC	CGATGTTCAACTTGCCTGTGTAG
S1pr2	ATGGGCGGCTTATACTCAGAG	GCGCAGCACAAGATGATGAT
S1pr3	ACTCTCCGGGAACATTACGAT	CAAGACGATGAAGCTACAGGTG
S1pr4	GTCAGGGACTCGTACCTTCCA	GATGCAGCCATACACACGG
S1pr5	GCTTTGGTTTGCGCGTGAG	GGCGTCCTAAGCAGTTCCAG

### 2.7 Biochemical assays

Platelet poor plasma was isolated by first centrifuging 500-800 μL of citrated blood for 20 minutes at 4,000 x g. The supernatant was transferred to a new Eppendorf tube and spun down at 12,000 rpm for 2 minutes. The clear supernatant was then aliquoted to new tubes for analysis or storage. Approximately 100 μL of plasma was used for analysis of alanine aminotransferase (ALT) levels using VetScan VS2 (Abaxis, Union City, CA).

### 2.8 Statistical and data analyses

Graphs and statistical analyses were done using GraphPad Prism version 9 for Windows (GraphPad Software, La Jolla, CA; www.graphpad.com). Data are presented as mean ± S.E.M in each graph. Each dot in the dot plot represents one mouse or biological replicate. The Grubb’s test (extreme studentized deviant method) was used to identify outliers to determine if the most extreme value in a data set is a significant outlier from the rest. For statistical comparisons of multiple experimental groups against the control group, parametric data were analyzed by ANOVA and the Tukey’s Multiple Comparison method was employed to determine which pairs of means are different. A p-value of < 0.05 was considered significant.

## 3 Results

### 3.1 Etrasimod and Amiselimod treatment reduce liver injury in NASH

**Figure 1:**
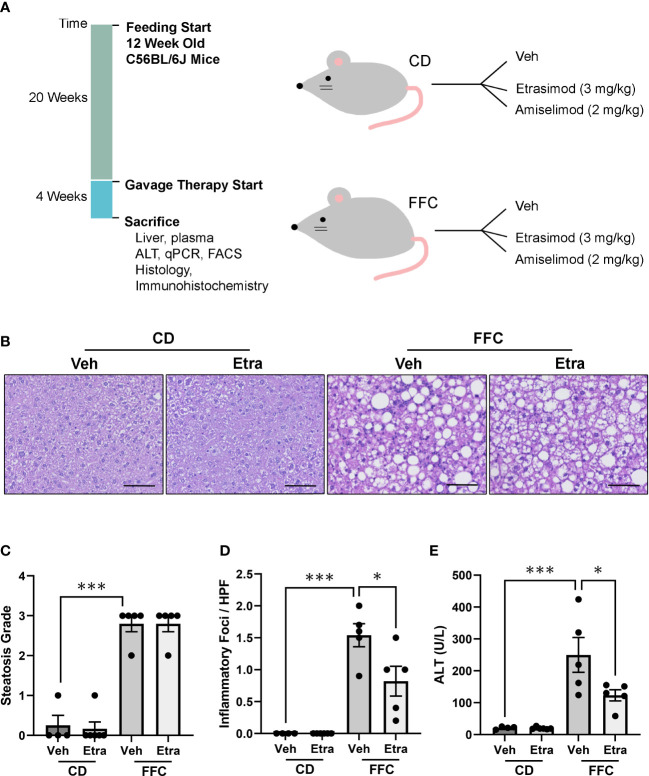
Etrasimod treatment reduces liver injury. **(A)** Timeline of mouse study and analysis after euthanasia. Dietary feeding started 6 weeks after acquisition of C57BL/6J male mice at 12 weeks of age. Mice were randomized to receive standard CD or FFC diet for 24 weeks. At 20 weeks of feeding, CD and FFC cohorts received vehicle or Etrasimod and vehicle or Amiselimod drug treatment for 4 weeks. Mice were euthanized at the end of gavage therapy and processed for analysis. **(B)** Representative liver histology shown by Hematoxylin and Eosin (H&E) staining of vehicle or Etrasimod treated mice in CD and FFC cohorts. Scale bar equals 50 μm. **(C)** Steatosis component of the NAFLD Activity Score for mouse cohorts treated with vehicle or Etrasimod. Each mouse is graded for steatosis (0–3). Less than 5% steatosis = 0, 5~33% = 1, 34~66% = 2, >66% = 3. Each dot represents one biological replicate. **(D)** The inflammatory (0-3) component score in NAS grading is shown for CD and FFC cohorts treated with vehicle or Etrasimod. If there are no inflammatory foci = 0, <2 inflammatory foci = 1, 2~4 inflammatory foci = 2, >4 inflammatory foci = 3. **(E)** Plasma ALT levels for CD and FFC cohorts treated with vehicle or Etrasimod at completion of the study. CD (n=10) and FFC (n=10). *p<0.05, ***p<0.001.

A dietary NASH mouse model was generated by feeding mice a FFC diet for 24 weeks. Next, both CD (control) and FFC-fed groups were randomized to receive Etrasimod (3 mg/Kg) or vehicle, or Amiselimod (2 mg/Kg) or vehicle treatment based on effective doses reported in the literature (**Figure 1A**) (12, 13). The liver histology from FFC-fed mice showed a significant increase in steatosis and inflammation compared to those mice that received CD (**Figure 1B**). Steatosis and inflammatory foci were quantified using the NAFLD activity score, which showed that Etrasimod treatment did not reduce steatosis in FFC-fed mice (**Figure 1C**). Similarly, Amiselimod treatment did not impact steatosis (**Supplementary Figures 1A, B**). On the other hand, Etrasimod treatment significantly reduced inflammatory foci on histological examination of the FFC group (**Figure 1D**). Correspondingly, the ALT level was reduced with Etrasimod treatment in the FFC-fed group (**Figure 1E**), demonstrating improvement in liver inflammation and injury in FFC-fed mice. In contrast, Amiselimod treatment did not reduce inflammatory foci though lowered ALT levels (**Supplementary Figures 1C, D**).

### 3.2 Metabolic parameters are unchanged in mice treated with Etrasimod or Amiselimod

**Figure 2:**
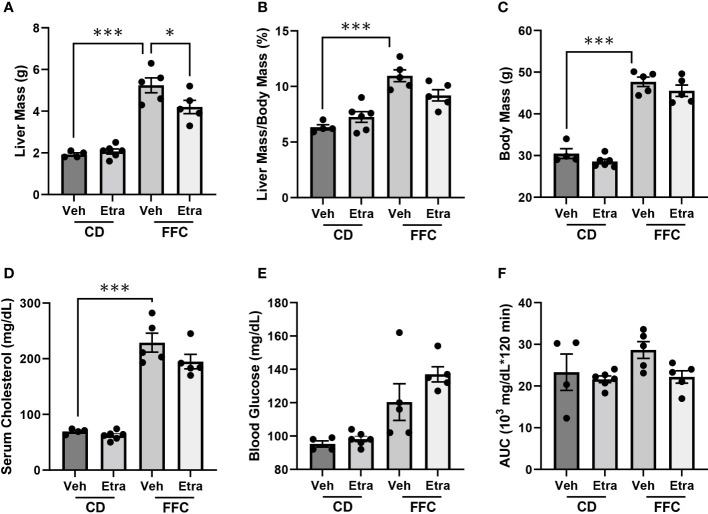
Metabolic characterization of CD and FFC-fed mice with Etrasimod treatment. **(A)** Liver mass, **(B)** liver to body mass ratio, **(C)** and body mass of groups treated with vehicle or Etrasimod. **(D)** Total cholesterol and **(E)** fasting blood glucose with vehicle or Etrasimod treatment. **(F)** Area under the curve (AUC) for the glucose tolerance test at 22 weeks on diet and 2 weeks of Etrasimod treatment. CD (n=10) and FFC (n=10). *p<0.05, ***p<0.001.

CD-fed and FFC-fed mice maintained comparable body mass, liver mass and relative liver mass (**Figures 2A–C**, **Supplementary Figures 2A–C**), regardless of Etrasimod or Amiselimod treatment, confirming that the improvement in liver injury and inflammation were not due to do a lack of weight gain. Etrasimod administration did not lower the FFC diet-induced increase of cholesterol (**Figure 2D**). There was a trend toward increase in fasting blood sugar in vehicle-treated FFC-fed mice; however, levels were similar to Etrasimod-treated FFC-fed mice (**Figure 2E**). Neither FFC-diet induced hypercholesterolemia nor elevated fasting blood sugars were lowered following Amiselimod administration (**Supplementary Figures 2D, E**). FFC-fed Etrasimod and Amiselimod treated mice demonstrated comparable glucose disposal to vehicle treated mice following intraperitoneal glucose challenge (**Figure 2F**, **Supplementary Figure 2F**).

### 3.3 Etrasimod treatment leads to changes in intrahepatic leukocyte populations

**Figure 3:**
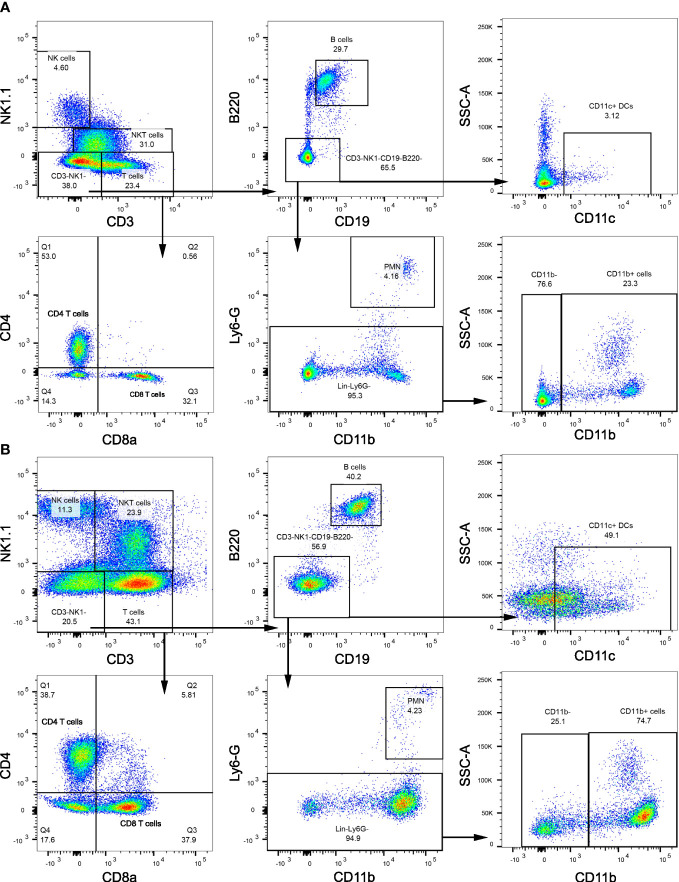
Flow cytometry gating strategy. **(A)** Flow cytometry gating strategy that is used to isolate immune populations within the liver of CD-fed mice and **(B)** FFC-fed mice. Immune populations were identified as follows: NK cells (CD3^-^NK1.1^+^), NKT cells (CD3^+^NK1.1^+^), B cells (CD3^-^NK1.1^-^CD19^+^B220^+^), DCs (CD3^-^NK1.1^-^CD19^-^B220^-^CD11c^+^), PMNs (CD11b^+^Ly6G^-^), CD11b^+^ myeloid cells (CD11b^+^ Ly6G^-^). T cells (CD3^+^NK1.1^-^) were selected for further analysis to identify CD4 T cells (CD3^+^NK1.1^-^CD4^+^CD8a^-^), CD8 T cells (CD3^+^NK1.1^-^CD4^-^CD8a^+^) and DNT cells (CD3^+^NK1.1^-^CD4^-^CD8a^-^).

**Figure 4:**
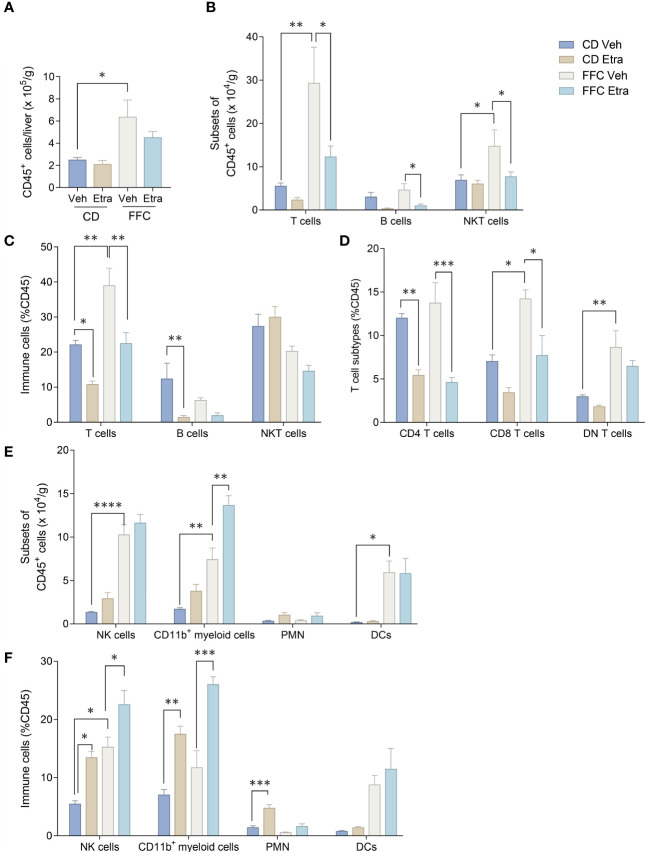
Etrasimod treatment alters intrahepatic leukocyte populations. **(A)** Number of CD45^+^ cells per gram acquired for FACS analysis in CD and FFC cohorts. **(B)** Subsets of CD45^+^ cells, **(C)** immune cell profiling expressed in %CD45 cells that are T cells, B cells and NKT cells by FACS analysis in livers from CD and FFC cohorts that received vehicle or Etrasimod treatment. **(D)** T-cell subtypes expressed in %CD45 cells that are CD4 positive, CD8 positive or double negative in CD and FFC cohorts. **(E)** Subsets of CD45^+^ cells that are NK cells, CD11b^+^ myeloid cells, PMNs and DCs. **(F)** Immune cells expressed in %CD45 that are NK cells, CD11b^+^ myeloid cells, PMNs and DCs. CD (vehicle n=4, Etrasimod n=6) and FFC (vehicle n=5, Etrasimod n=5). *p<0.05, **p<0.01, ***p<0.001, ****p<0.0001.

We next examined the changes in intrahepatic leukocyte populations in FFC-fed mice treated with Etrasimod or Amiselimod by flow cytometry of the intrahepatic leukocyte populations. After isolation, viable cells were selected for further analysis (**Supplementary Figure 3**). Our flow cytometry panel was designed to characterize the major immune cell populations within the liver (**Figure 3**). As expected, the FFC-diet led to an increase in the number of total CD45^+^ cells (**Figure 4A**), T cells, and NKT cells (**Figures 4B, C**) in the liver. Etrasimod treatment led to a reduction in the frequency of T and B cells in the liver of CD- and FFC-fed mice (**Figures 4B, C**). CD3^+^ NK1.1^+^ NKT cells were also reduced in FFC-fed mice (**Figures 4B, C**), though they remained unchanged in CD-fed mice. Further analysis of T-cell subsets demonstrated no change in CD4^+^ T cells in FFC-fed mice compared to CD-fed mice, though CD4^+^ T cells were reduced by Etrasimod in both CD-fed and FFC-fed mice (**Figure 4D**). CD8^+^ T cells were increased in FFC-fed mice in comparison with CD-fed mice and were decreased following Etrasimod administration. We observed an increase in double negative (DN) T cells in FFC-fed mice which were unchanged following Etrasimod administration in FFC-fed mice, though reduced by Etrasimod in CD-fed mice. Etrasimod increased the frequency and percentage of NK cells in the liver in CD-fed mice, but not in FFC-fed mice (**Figures 4E, F**). The frequency and percentage of CD11b^+^ cells, which includes myeloid cells and polymorphonuclear cells (PMNs), were increased in both CD-fed and FFC-fed Etrasimod-treated mice. In contrast, the total number of CD45^+^ cells in the liver were unaffected in either CD-fed or FFC-fed mice treated with Amiselimod (**Supplementary Figure 4A**). Intrahepatic leukocyte populations in Amiselimod treated CD-fed mice had changes similar to Etrasimod with a reduction in T cells and B cells and an increase in CD11b^+^ myeloid cells; however, these effects were more modest in FFC-fed mice suggesting that, at the dose tested, Amiselimod was less effective than Etrasimod in modulating the distribution of immune cells under conditions of nutrient excess (**Supplementary Figure 4**). Though T cells were not reduced in FFC-fed mice treated with Amiselimod, the T cell subsets were altered like Etrasimod.

### 3.4 Macrophage accumulation and expression of proinflammatory markers are reduced with drug treatment

**Figure 5:**
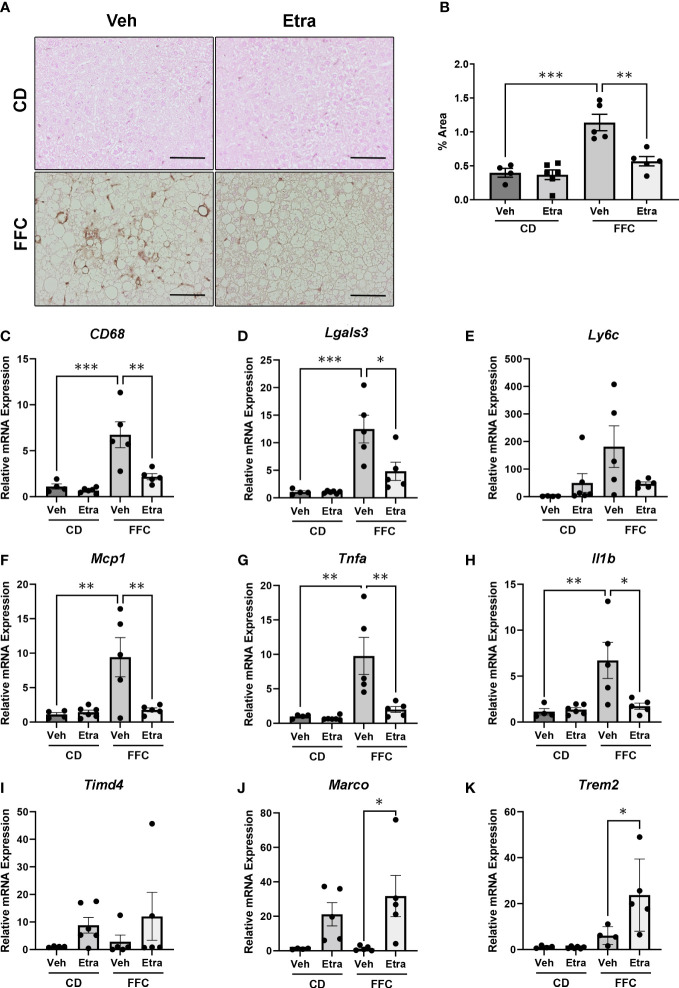
Macrophage accumulation and inflammatory markers are reduced in Etrasimod treated mice. **(A)** Representative images showing immunohistochemistry for *Mac-2* in liver tissue sections for vehicle and Etrasimod treated mice in both CD and FFC cohorts. Scale bar equals 50 μm. **(B)** Mac-2 staining was quantified as percentage of positive immunoreactive area in CD and FFC-fed mice treated with vehicle or Etrasimod. **(C–E)** Relative mRNA expression of *Cd68, Lgals3* and *Ly6c* in CD and FFC cohorts treated with vehicle or Etrasimod. **(F–H)** Relative mRNA expression of monocyte chemoattractant protein-1 *(Mcp1)*, tumor necrosis factor alpha (*Tnfa)* and interleukin-1 beta (*Il1b)* in liver tissues of CD and FFC-fed mice treated with vehicle or Etrasimod. **(I–K)** Relative mRNA expression of *Timd4, Marco* and *Trem2* in CD and FFC cohorts treated with vehicle or Etrasimod. CD (vehicle n=4, Etrasimod n=6) and FFC (vehicle n=5, Etrasimod n=5). *p<0.05, **p<0.01, ***p<0.001.

NASH is characterized by intrahepatic macrophage accumulation. As our flow cytometry panel did not include markers to differentiate between infiltrating monocyte-derived and tissue-resident macrophages, we assessed their accumulation and activation using immunohistochemistry and gene expression analysis. Immunohistochemistry for Mac-2/Galectin-3 in liver sections showed that activated macrophage accumulation was visibly reduced in livers of both Etrasimod and Amiselimod treated mice (**Figure 5A**, **Supplementary Figure 5A**). Quantification of *Mac-2/Galectin-3* positive areas confirmed significant decrease in macrophage accumulation in Etrasimod (**Figure 5B**) and Amiselimod (**Supplementary Figure 5B**) treated mice. There was also a significant reduction in relative mRNA expression of macrophage markers *CD68* and *Lgals3* (**Figures 5C, D**). Similar changes were observed in *Ly6c*, a marker of proinflammatory monocytes, though this did not achieve statistical significance (**Figure 5E**). By extension, we asked whether drug treatment had an effect on levels of proinflammatory chemokines and cytokines in the liver. Relative mRNA expression of monocyte chemotactic protein 1 (*Mcp1*), tumor necrosis factor alpha (*Tnfa)* and interleukin 1 beta (*Il1b)* were significantly reduced with both Etrasimod (**Figures 5F–H**) and Amiselimod (**Supplementary Figures 5F–H**) treatment in FFC mice. Thus, Etrasimod and Amiselimod treatment not only reduced inflammatory macrophage accumulation in the liver, but also reduced levels of proinflammatory cytokines that aggravate the inflammatory response. In Etrasimod treated FFC-fed livers the resident macrophage marker *Timd4* (**Figure 5I**) was not upregulated, unlike Amiselimod (**Supplementary Figure 5I**). We also observed an increase in *Marco*, a marker of non-inflammatory macrophage populations (**Figure 5J**, **Supplementary Figure 5J**) and the lipid associated macrophage marker *Trem2* (**Figure 5K**, **Supplementary Figure 5K**). These data suggest that the increase in CD11b observed by flow cytometry may be due to an increase in the abundance of resident and non-inflammatory macrophages.

### 3.5 Palmitate treatment induces the expression of S1P_1_ and S1P_2_ in macrophages

**Figure 6:**
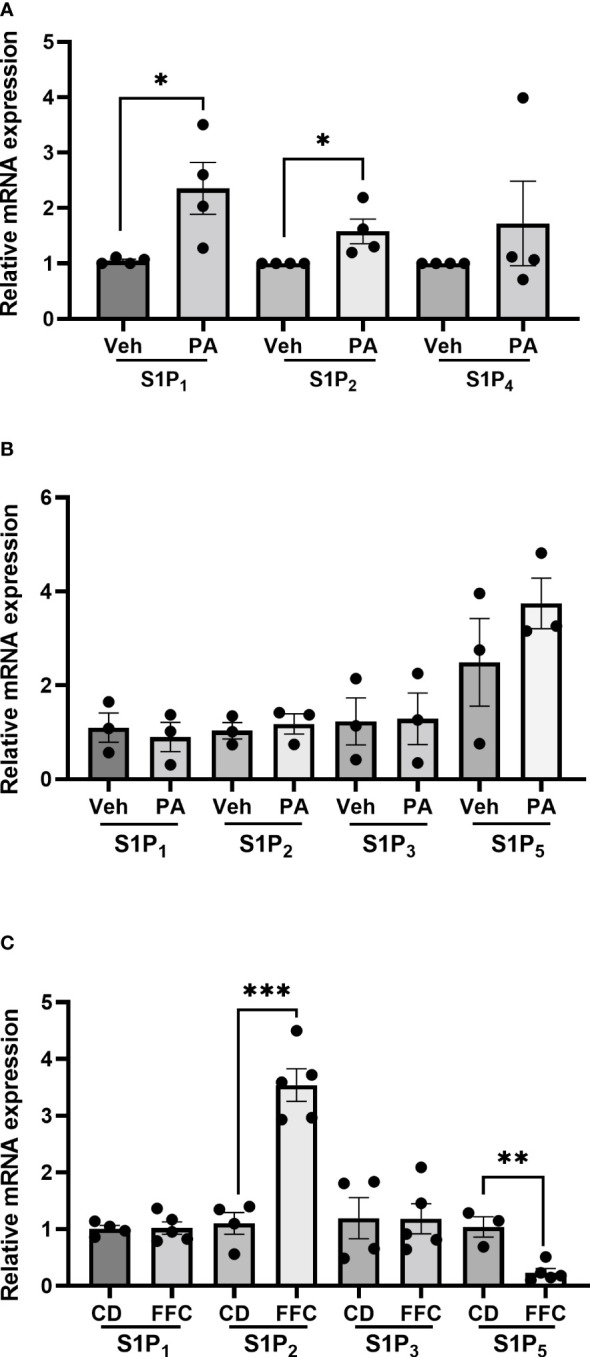
S1P_1_ and S1P2 are upregulated in palmitate-treated BMDM. **(A)** In BMDM, expression of S1P_1_ and S1P_2_ mRNA was induced following palmitate treatment whereas the abundance of S1P_4_ remained unchanged (vehicle n=4, palmitate n=4). **(B)** In PMH, S1P_1_, S1P_2_, S1P_3_ and S1P_5_ were detected and remained unchanged with palmitate treatment (vehicle n=3, palmitate n=3). **(C)** In whole livers, S1PR_2_ was upregulated, S1PR_5_ was downregulated, and no change was noted in S1PR_1_ and S1PR_3_ in mice fed the FFC diet in comparison to CD fed controls. CD (n=4) and FFC (n=5). *p<0.05, **p<0.01, ***p<0.001.

To understand whether the differential effects of Etrasimod and Amiselimod may be mediated by variations in the expression of S1P receptors, the reported expression of S1P receptors in hepatocytes, bone marrow monocytes and macrophages was compared from a publically available single cell RNA sequencing data (19) (https://www.czbiohub.org/tabula-muris/), **Table 2**. In this dataset S1P_4_ mRNA was most abundant in macrophages and monocytes, and S1P_1_ was most abundant in hepatocytes. Next, BMDM and PMH were treated with palmitate to induce lipotoxic stress (10). In BMDMs, the expression of S1P_1_ and S1P_2_ mRNA was induced following palmitate treatment, whereas the abundance of S1P_4_ remained unchanged (**Figure 6A**). S1P_1_, S1P_2_, S1P_3_ and S1P_5_ were detected in hepatocytes and remained unchanged with palmitate treatment (**Figure 6B**). In contrast to isolated cells, analysis of the abundance of S1P receptors in whole livers demonstrated no change in S1PR_1_ and S1PR_3_, upregulation of S1PR_2_, and downregulation of S1PR_5_ in mice fed the FFC diet in comparison to CD fed controls (**Figure 6C**). As S1P_1_ was upregulated in BMDM by lipotoxic stress without any induction in PMH or whole livers, this may be the primary target on macrophages for the beneficial effects of Etrasmiod and Amiselimod. Further examination of hepatocellular proliferation demonstrated low basal Ki67 positivity, consistent with the literature (20). Ki67 positivity was increased in FFC-fed vehicle treated livers, as has been reported in mouse models of NASH (21, 22) and reduced following treatment with Etrasimod and Amiselimod (**Supplementary Figure 6**). Hepatocellular expression of HNF4α was comparably reduced in vehicle treated and Etrasimod or Amiselimod treated FFC-fed mouse livers (**Supplementary Figure 7**).

**Table 2:** S1P_1-5_ Mean expression ln(1+CPM) in hepatocytes, macrophages, and monocytes.

S1P Receptors	Hepatocytes	Macrophage	Monocyte
S1P_1_	2.44	0.18	0.17
S1P_2_	0.31	0.01	0.59
S1P_3_	0.01	0.04	0.04
S1P_4_	0.02	2.45	1.94
S1P_5_	0.30	0.01	0.29

(https://www.czbiohub.org/tabula-muris/).

## 4 Discussion

S1P is a bioactive sphingolipid with pleiotropic receptor-mediated signaling responses in diverse physiological and pathophysiological contexts. S1P dysregulation and subsequent mediation of lymphocyte trafficking is implicated in chronic sterile inflammatory diseases, including NASH. Here we have explored the therapeutic efficacy of Etrasimod, a selective S1P receptor 1, 4, and 5 modulator in comparison with Amiselimod, a selective S1P receptor 1 modulator, in attenuating murine NASH and associated intrahepatic leukocyte populations via antagonism of the S1P signaling axis. We report that: 1) S1P receptor antagonism attenuates liver injury, ii) Etrasimod reduces liver inflammatory infiltrates including T cells, B cells, and NKT cells, and iii) both drugs reduce inflammatory macrophage accumulation in a dietary model of murine NASH. These data establish a mechanistic link between S1P/S1P receptor signaling and the immune inflammatory response in NASH, as selective inhibition of S1P receptors demonstrated reversal of adverse features of NASH.

S1P is a signaling sphingolipid that participates in specific receptor signaling by acting on five GPCRs linked to intracellular signaling pathways (12). Dysregulation of S1P concentration is known to contribute to pathological conditions and inflammatory diseases including NASH (4). Mechanistically, toxic lipid species, such as palmitate, involved in hepatic lipotoxicity serve as precursors for downstream sphingolipids including S1P. In our previous study, we demonstrated that pharmacological treatment with Fingolimod (FTY720) effectively reversed murine NASH including reduction in ALT, inflammation, and hepatic inflammatory macrophage accumulation (10). We observed similar effects on liver injury with Etrasimod and Amiselimod. The contribution of various intrahepatic immune cell populations, including macrophages, T cells, and B cells, is being increasing appreciated in NASH (1). Having previously characterized liver injury and inflammation, our objective in this study was to examine changes in intrahepatic immune cells in NASH and how they are modified following inhibition of the S1P receptor-mediated signaling axis.

There is evidence to support that S1P receptor modulation impairs trafficking of macrophages, T cells, and B cells, all of which are implicated in NASH pathogenesis (1, 10, 23). Therefore, we examined the changes in intrahepatic leukocyte composition in mice treated with Etrasimod and Amiselimod. We found that Etrasimod treatment led to specific shifts in leukocyte populations including reduction in T cells, B cells, and NKT cells in FFC-fed mice and an increase in CD11b^+^ myeloid cells and PMNs in both CD and FFC-fed mice. The reduction in T cell and B cell egress from lymphoid tissues is a well characterized effect of S1P antagonism, and our findings are consistent with this mechanism of action (24, 25). In contrast Amiselimod induced similar changes in CD-fed mice; however, this effect was prevented in mice with dietary NASH suggesting perhaps that Amiselimod was less effective under conditions of nutrient stress. These data suggest that Etrasimod, which antagonizes 1, 4, and 5, versus Amiselimod which antagonizes S1P receptor 1 alone, was more effective likely due to effects on multiple proinflammatory immune cell types, though we cannot exclude a dose-dependent effect. DNT cells are a subpopulation of regulatory T cells known to suppress immune responses in both mice and humans (26). The increase in DNT cells may contribute to the suppressive properties of Etrasimod in the immune response of NASH. The specific contributions and interrelatedness of these cell types will require further investigation aided by the incorporation of lineage specific knockout mice, for example, macrophage-specific, T cell-specific, and B cell-specific knockouts. Nonimmune cells such as hepatocytes and sinusoidal endothelial cells also express S1P receptors. A reduction in hepatocyte proliferation was noted in Etrasimod and Amiselimod treated livers, which could be a direct effect of the inhibitors or an indirect effect due to a reduction in inflammation. Examination of sinusoidal endothelial cells is beyond the scope of the current manuscript.

Our flow cytometry panel only included CD11b to quantify myeloid cells. Therefore, we employed a combination of immunohistochemistry and gene expression to understand the increase in CD11b^+^ myeloid cells observed with Etrasimod, based on recent and established markers of macrophage subsets. Histologically, proinflammatory infiltrating macrophage accumulation, measured by immunohistochemistry and qPCR for galectin-3 (*Lgals3*) was increased in FFC-fed liver, consistent with our previous findings (10), and was lowered in Etrasimod treated FFC-fed mouse livers (27–29). Examination of markers of resident hepatic macrophages (*Timd4*), non-inflammatory macrophages (*Marco*), and lipid-associated macrophages (*Trem2*) suggested an increase in macrophage subsets which could explain the increase in CD11b^+^ myeloid cells (30, 31). These data suggest a differential response of macrophage subsets to S1P receptor antagonism *in vivo*, potentially due to only minimal inhibition of S1P_5_ by Etrasimod as S1P_5_ is known to promote egress of monocytes from the bone marrow (32). This testable hypothesis will be approached in future studies with myeloid-specific S1P_1_ knockout mice. Since chemokines and cytokines are secreted by multiple cell types, their reduction likely reflects overall lower immune cell infiltration in the livers of mice treated with Etrasimod and Amiselimod.

Etrasimod is an S1P receptor modulator with antagonistic functional activity against S1P_1_ and partially against S1P_4_ and S1P_5_. Etrasimod has no activity towards S1P_2_ or S1P_3_ (33). S1P_1_ is expressed on major immune cell types including: macrophages, neutrophils, dendritic cells, monocytes, NK cells, T cells, and B cells where they participate in recruitment and trafficking (25). S1P_4_ is widely expressed on dendritic cells, neutrophils, macrophages, monocytes, T cells, and B cells; recent findings suggest its roles in cell differentiation, recruitment, and migration. Patrolling monocytes and NK cells both express high levels of S1P_5_ (34). S1P_2_ is expressed on B cell, monocytes, eosinophils, and mast cells and S1P_3_ on T cells, B cells, macrophage, monocytes, neutrophils, eosinophils, mast cells and dendritic cells. The present study revealed that receptor-specific modulation by Etrasimod reduces accumulation of macrophages in the liver and reduces intrahepatic T cells, B cells and NKT cells in FFC-fed mice, which would be consistent with the cell-specific pattern of expression of S1P receptors and the antagonistic activity of Etrasimod. T cells are known to egress from secondary lymphoid organs to the lymphatic and blood circulation through S1P/S1P_1_ receptor trafficking (34, 35). The antagonistic activity of Etrasimod on S1P_1_ receptors may have suppressed T cell egress from the thymus and secondary lymphoid organs, resulting in fewer infiltrating T cells, similar to effects in ulcerative colitis (36). In patients with relapsing multiple sclerosis, administration of low dose FTY720 and subsequent blockage of S1P-mediated T cell egress has shown beneficial disease outcomes and fewer side effects (37). On the other hand, B cells express all 5 S1P receptors, yet were reduced by Etrasimod administration suggesting a predominant role for S1P_1, 4, 5_ in B cells. B cell reduction could also be a result of inhibiting S1P-mediated lymphocyte trafficking through Etrasimod, repressing lymphocyte migration to peripheral tissues. Though S1P_1_ is more ubiquitously expressed by immune cells, at the doses we tested, Etrasimod was more effective than Amiselimod at skewing the distribution of immune cells under nutrient stress conditions, suggesting redundant roles for each receptor subtype.

In conclusion, we demonstrate that modulating S1P_1_, S1P_4_ and S1P_5_ by Etrasimod treatment ameliorates cardinal features associated with progressive NASH. Additional studies are required to investigate specific S1P receptor signaling on each immune cell type and their mechanistic roles in leukocyte trafficking and subsequent biological outcomes in liver inflammation in NASH. We cannot attribute specific outcomes to any individual S1P receptor or a specific cell type; yet, antagonizing multiple receptors appears to have a greater therapeutic effect than S1P_1_ alone. These data provide an interesting insight into the effect of S1P receptor modulation on the immune cell repertoire and its potential therapeutic benefits in NASH. Notably, FTY720 is approved for the treatment of multiple sclerosis and ozanimod is approved for ulcerative colitis. Consistent with these, Etrasimod and Amiselimod have been used in clinical trials for ulcerative colitis and multiple sclerosis, respectively (11, 38). Future studies are required to evaluate the efficacy of Etrasimod and Amiselimod in treating human NASH.

## Data availability statement

The original contributions presented in the study are included in the article/**Supplementary Material**. Further inquiries can be directed to the corresponding author.

## Ethics statement

The animal study was reviewed and approved by Institutional Animal Care and Use Committee (IACUC).

## Author contributions

C-YL, NV, AM, XR, YN, TS, DD, MS, FB conducted experiments. C-YL, AM, XR analyzed and graphed data. HM designed, supervised, and analyzed experiments. C-YL and HM prepared figures and drafted the manuscript. All authors contributed to the article and approved the submitted version.

## Supplementary material

Supplementary °C Amiselimod treatment reduces liver injury. **(A)** Liver histology shown by H&E staining of vehicle or Amiselimod treated mice in CD and FFC cohorts. Scale bar equals 50 μm. **(B)** Steatosis component of the NAFLD Activity Score (NAS) for CD and FFC mouse cohorts treated with vehicle or Amiselimod. Each mouse is graded for steatosis (0-3). Less than 5% steatosis = 0, 5~33% = 1, 34~66% = 2, >66% = 3. Each dot represents one biological replicate. **(C)** The inflammatory (0-3) component score in NAS grading is shown for CD and FFC cohorts treated with vehicle or Amiselimod. If there are no inflammatory foci = 0, <2 inflammatory foci = 1, 2~4 inflammatory foci = 2, >4 inflammatory foci = 3. **(D)** Plasma ALT levels for CD and FFC cohorts treated with vehicle or Amiselimod at completion of the study. CD (n=10) and FFC (n=10). *p<0.05, **p<0.01, ***p<0.001

Supplementary Figure 2 Metabolic characterization of CD and FFC-fed mice with Amiselimod treatment. **(A)** Liver mass, **(B)** liver to body mass ratio and **(C)** body mass of CD (n=9) and FFC (n=11) groups treated with vehicle or Amiselimod. **(D)** Total cholesterol and **(E)** fasting blood glucose with vehicle or Amiselimod treatment. CD (n=10) and FFC (n=7). **(F)** AUC for the glucose tolerance test at 22 weeks on diet and 2 weeks of Amiselimod treatment. CD (n=10) and FFC (n=10). ***p<0.001

Supplementary Figure 3 Viability plot for flow cytometry. **(A)** Representative image shows viability of samples using live/dead cells counts by employing Zombie Aqua dye.

Supplementary Figure 4 Amiselimod treatment alters some intrahepatic leukocyte populations. **(A)** Number of CD45^+^ cells per gram acquired for FACS analysis in CD and FFC cohorts. **(B)** Subsets of CD45^+^ cells and **(C)** immune cells expressed in %CD45 that are T cells, B cells and NKT cells by FACS analysis in livers from CD and FFC cohorts that received vehicle or Amiselimod treatment. Quantifications are expressed as %CD45 cells or total number. **(D)** T-cell subtypes expressed in %CD45 cells that are CD4 positive, CD8 positive or double negative in CD and FFC cohorts. **(E)** Subsets of CD45^+^ cells including NK cells, CD11b^+^ myeloid cells, PMNs and DCs and **(F)** immune cells expressed in %CD45 that are NK cells, CD11b^+^, PMNs and DCs. CD (vehicle n=4, Amiselimod n=5) and FFC (vehicle n=5, Amiselimod n=6). *p<0.05, **p<0.01, ***p<0.001.

Supplementary Figure 5 Macrophage accumulation and inflammatory markers are reduced in Amiselimod treated mice. **(A)** Representative images showing immunohistochemistry for *Mac-2* in liver tissue sections for vehicle and Amiselimod treated mice in both CD and FFC cohorts. Scale bar equals 50 μm. **(B)** Mac-2 staining was quantified as percentage of positive immunoreactive area in CD and FFC-fed mice treated with vehicle or Amiselimod. **(C-E)** Relative mRNA expression of *Cd68, Lgals3, Ly6c* in CD and FFC cohorts treated with vehicle or Amiselimod. **(F-H)** Relative mRNA expression of monocyte chemoattractant protein-1 *(Mcp1)*, Tumor necrosis factor alpha (*Tnfa)* and Interleukin 1 beta (*Il1b)* in liver tissues of CD and FFC-fed mice treated with vehicle or Amiselimod. **(I-K)** Relative mRNA expression of *Timd4, Marco* and *Trem2* positive macrophages in CD and FFC cohorts treated with vehicle or Amiselimod. CD (vehicle n=5, Amiselimod n=5) and FFC (vehicle n=5, Amiselimod n=6). *p<0.05, **p<0.01, ***p<0.001.

Supplementary Figure 6 FFC diet-induced increase in hepatocyte proliferation is reduced in Etrasimod and Amiselimod treated mice. **(A)** Representative images showing immunohistochemistry for Ki67 in liver tissue sections for vehicle and Etrasimod treated mice in both CD and FFC cohorts. **(B)** Positive immunoreactive staining (dark brown) was quantified as Ki67 positive nuclei per high power field in CD and FFC-fed mice treated with vehicle or Etrasimod. CD (vehicle n=4, Amiselimod n=6) and FFC (vehicle n= 5, Amiselimod n=6). **(C)** Representative images showing immunohistochemistry for Ki67 in liver tissue sections for vehicle and Amiselimod treated mice in both CD and FFC cohorts. **(D)** Positive immunoreactive staining (dark brown) was quantified as Ki67 positive nuclei per high power field in CD and FFC-fed mice treated with vehicle or Amiselimod. Scale bars represent 50 μm. CD (vehicle n=4, Amiselimod n=6) and FFC (vehicle n= 5, Amiselimod n=6). *p<0.05, **p<0.01, ***p<0.001.

Supplementary Figure 7 Hepatocyte differentiation is comparable in vehicle treated and Etrasimod or Amiselimod treated FFC-fed mouse livers. **(A)** Representative images showing immunohistochemistry for HNF4α in liver tissue sections for vehicle and Etrasimod treated mice in both CD and FFC cohorts. **(B)** Representative images showing immunohistochemistry for HNF4α in liver tissue sections for vehicle and Amiselimod treated mice in both CD and FFC cohorts. Scale bars represent 50 μm.
