# Supplementary figures and images for: Modulating sphingosine 1-phosphate receptor signaling skews intrahepatic leukocytes and attenuates murine nonalcoholic steatohepatitis
Source: Front Immunol. 2023 Apr 21;14:1130184. doi: 10.3389/fimmu.2023.1130184 (PMC10160388; doi:10.3389/fimmu.2023.1130184)

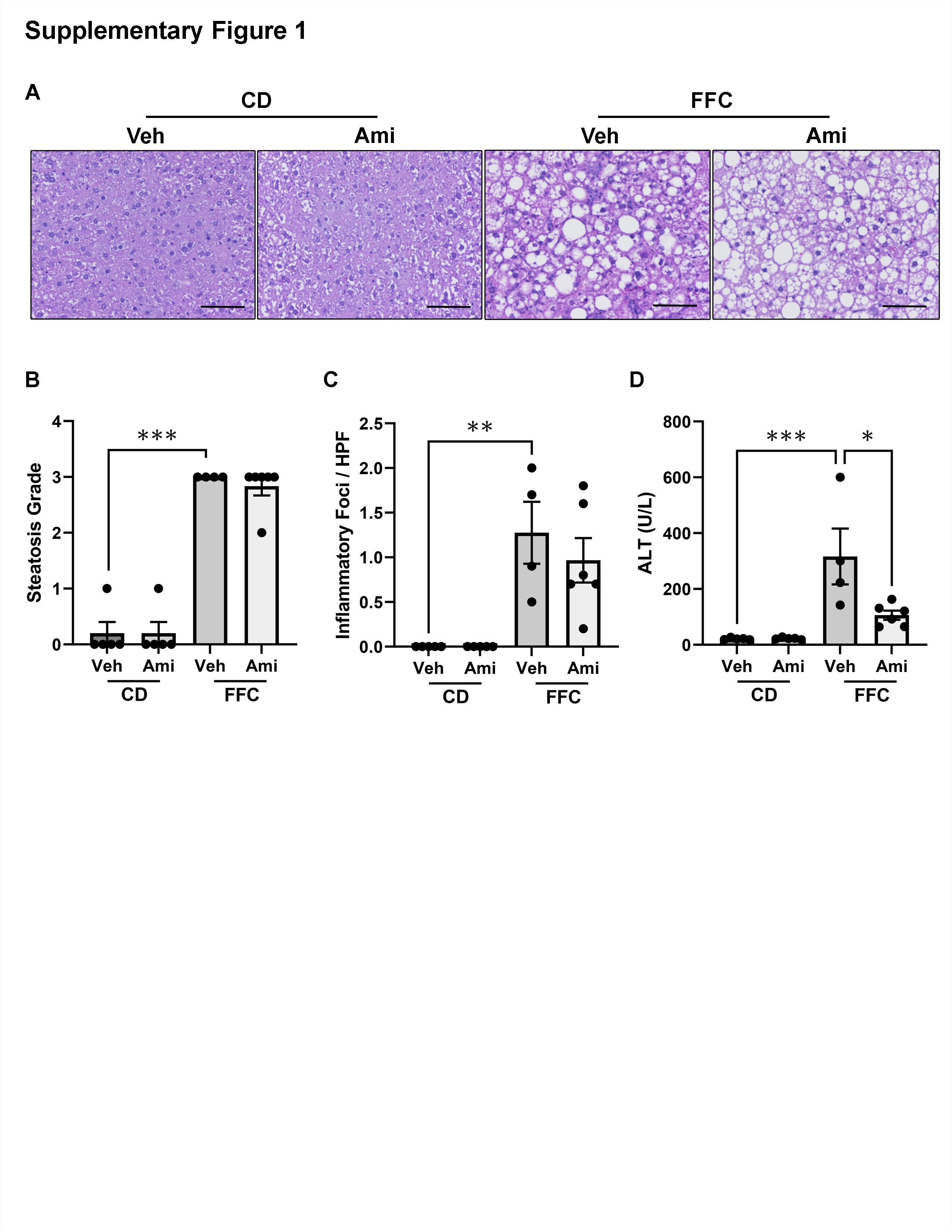

Supplement: Supplementary °C — Amiselimod treatment reduces liver injury. (A) Liver histology shown by H&E staining of vehicle or Amiselimod treated mice in CD and FFC cohorts. Scale bar equals 50 μm. (B) Steatosis component of the NAFLD Activity Score (NAS) for CD and FFC mouse cohorts treated with vehicle or Amiselimod. Each mouse is graded for steatosis (0-3). Less than 5% steatosis = 0, 5~33% = 1, 34~66% = 2, >66% = 3. Each dot represents one biological replicate. (C) The inflammatory (0-3) component score in NAS grading is shown for CD and FFC cohorts treated with vehicle or Amiselimod. If there are no inflammatory foci = 0, <2 inflammatory foci = 1, 2~4 inflammatory foci = 2, >4 inflammatory foci = 3. (D) Plasma ALT levels for CD and FFC cohorts treated with vehicle or Amiselimod at completion of the study. CD (n=10) and FFC (n=10). *p<0.05, **p<0.01, ***p<0.001 [file Image_1.tiff]

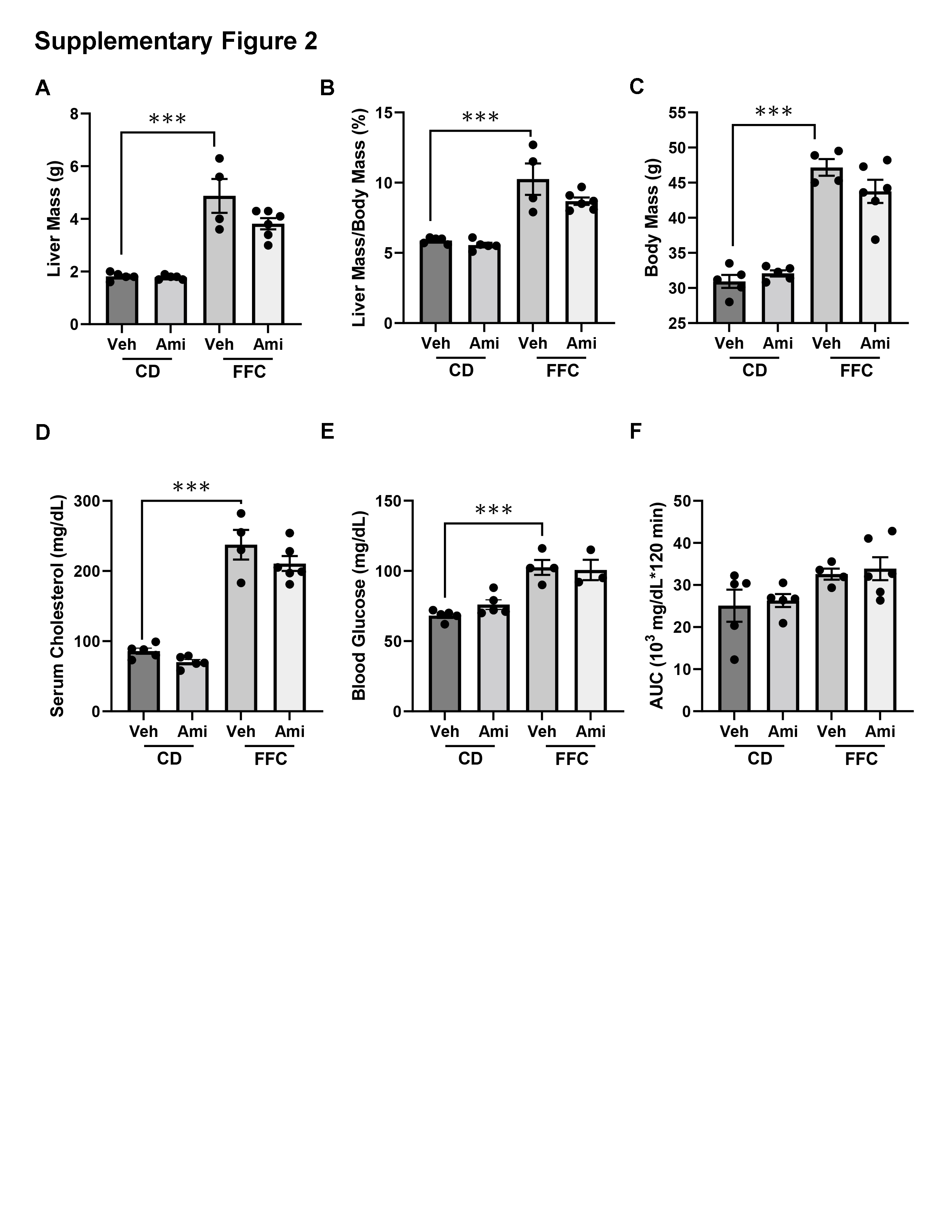

Supplement: Supplementary Figure 2 — Metabolic characterization of CD and FFC-fed mice with Amiselimod treatment. (A) Liver mass, (B) liver to body mass ratio and (C) body mass of CD (n=9) and FFC (n=11) groups treated with vehicle or Amiselimod. (D) Total cholesterol and (E) fasting blood glucose with vehicle or Amiselimod treatment. CD (n=10) and FFC (n=7). (F) AUC for the glucose tolerance test at 22 weeks on diet and 2 weeks of Amiselimod treatment. CD (n=10) and FFC (n=10). ***p<0.001 [file Image_2.tiff]

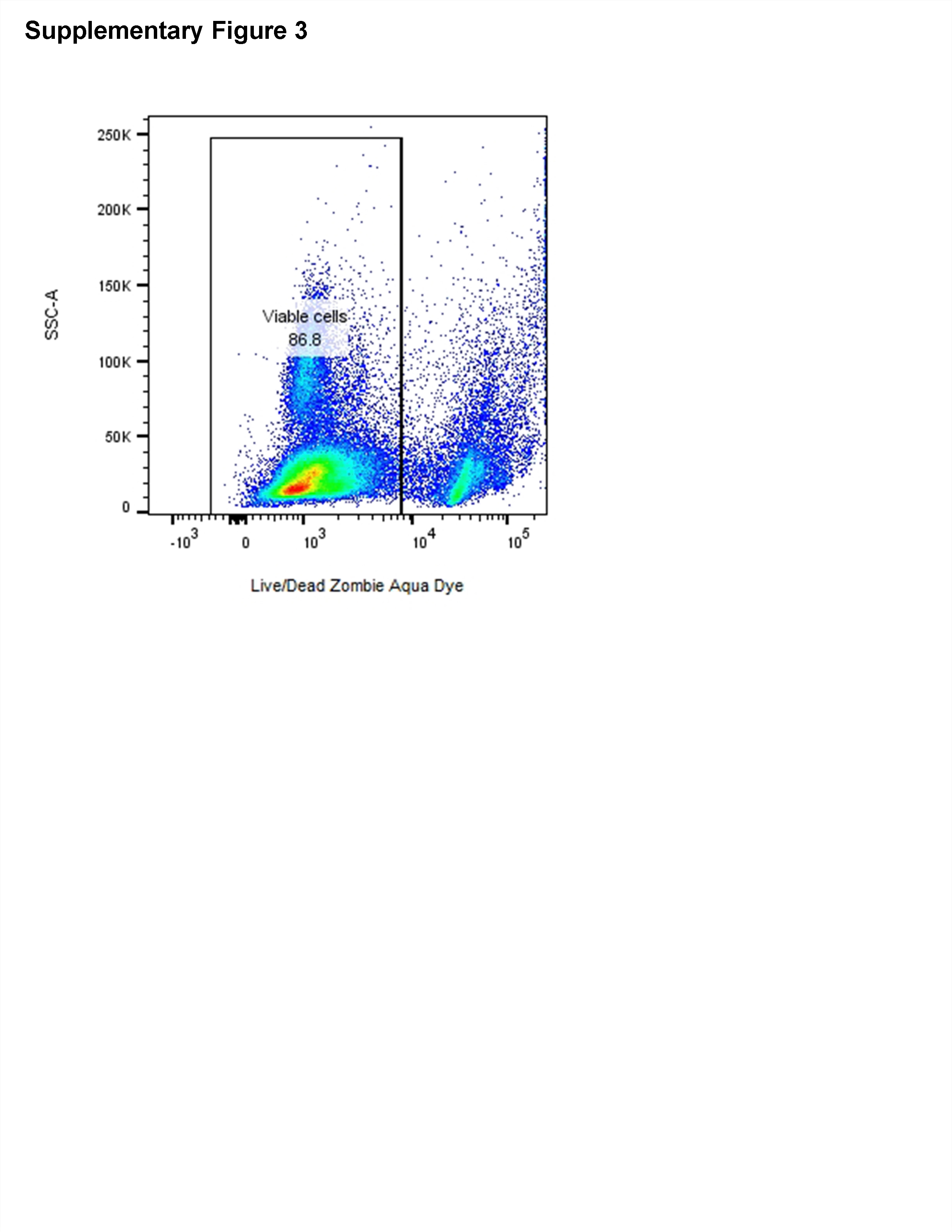

Supplement: Supplementary Figure 3 — Viability plot for flow cytometry. (A) Representative image shows viability of samples using live/dead cells counts by employing Zombie Aqua dye. [file Image_3.tiff]

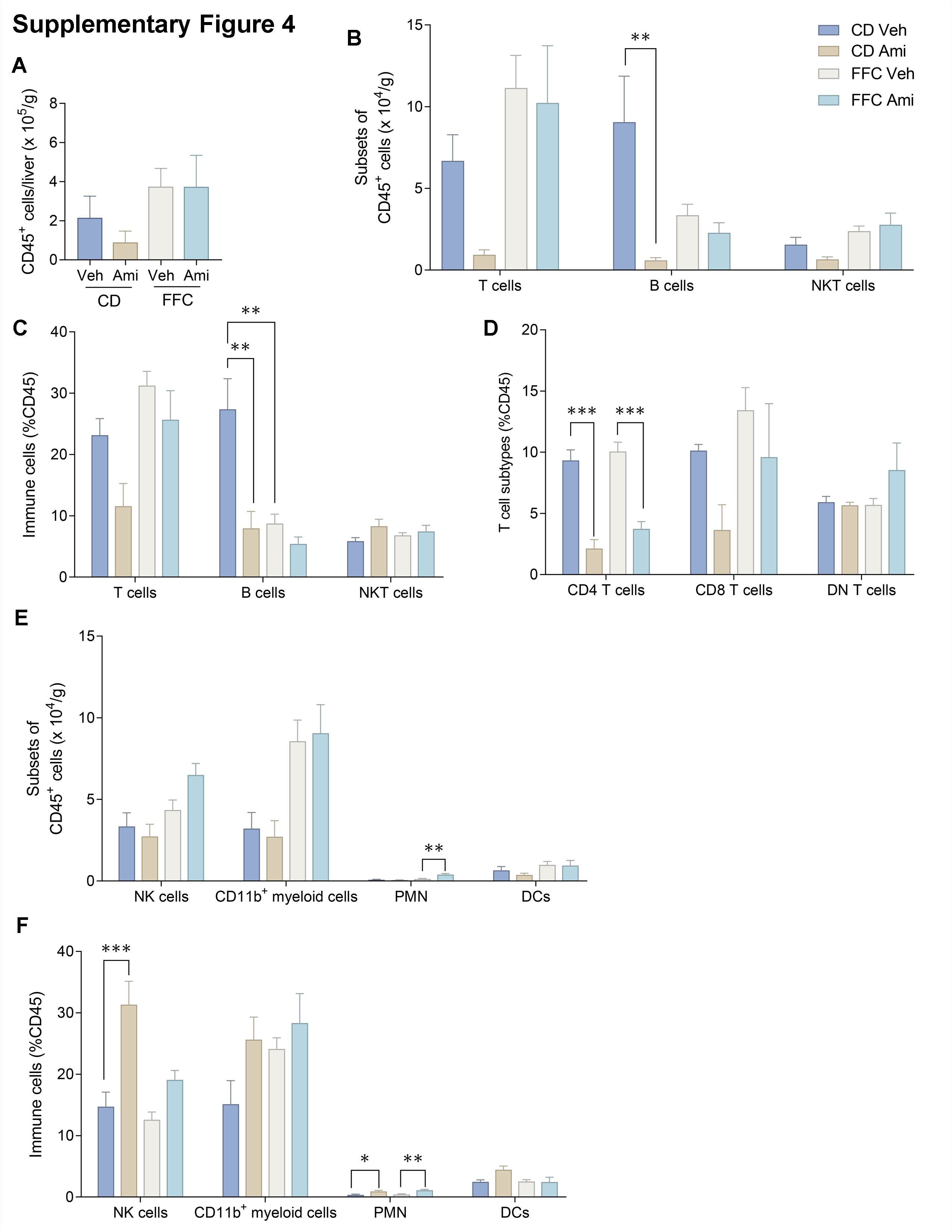

Supplement: Supplementary Figure 4 — Amiselimod treatment alters some intrahepatic leukocyte populations. (A) Number of CD45+ cells per gram acquired for FACS analysis in CD and FFC cohorts. (B) Subsets of CD45+ cells and (C) immune cells expressed in %CD45 that are T cells, B cells and NKT cells by FACS analysis in livers from CD and FFC cohorts that received vehicle or Amiselimod treatment. Quantifications are expressed as %CD45 cells or total number. (D) T-cell subtypes expressed in %CD45 cells that are CD4 positive, CD8 positive or double negative in CD and FFC cohorts. (E) Subsets of CD45+ cells including NK cells, CD11b+ myeloid cells, PMNs and DCs and (F) immune cells expressed in %CD45 that are NK cells, CD11b+, PMNs and DCs. CD (vehicle n=4, Amiselimod n=5) and FFC (vehicle n=5, Amiselimod n=6). *p<0.05, **p<0.01, ***p<0.001. [file Image_4.tiff]

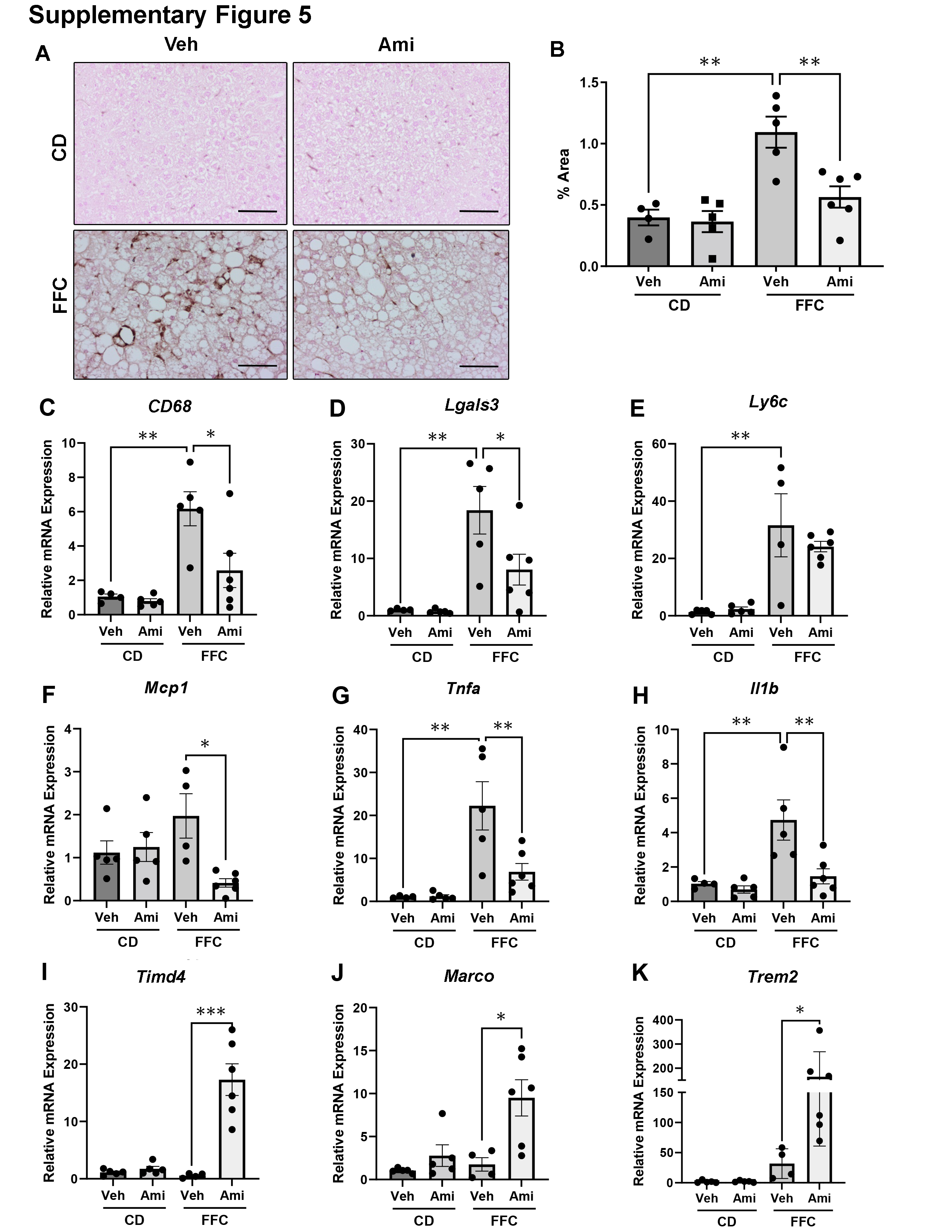

Supplement: Supplementary Figure 5 — Macrophage accumulation and inflammatory markers are reduced in Amiselimod treated mice. (A) Representative images showing immunohistochemistry for Mac-2 in liver tissue sections for vehicle and Amiselimod treated mice in both CD and FFC cohorts. Scale bar equals 50 μm. (B) Mac-2 staining was quantified as percentage of positive immunoreactive area in CD and FFC-fed mice treated with vehicle or Amiselimod. (C-E) Relative mRNA expression of Cd68, Lgals3, Ly6c in CD and FFC cohorts treated with vehicle or Amiselimod. (F-H) Relative mRNA expression of monocyte chemoattractant protein-1 (Mcp1), Tumor necrosis factor alpha (Tnfa) and Interleukin 1 beta (Il1b) in liver tissues of CD and FFC-fed mice treated with vehicle or Amiselimod. (I-K) Relative mRNA expression of Timd4, Marco and Trem2 positive macrophages in CD and FFC cohorts treated with vehicle or Amiselimod. CD (vehicle n=5, Amiselimod n=5) and FFC (vehicle n=5, Amiselimod n=6). *p<0.05, **p<0.01, ***p<0.001. [file Image_5.tiff]

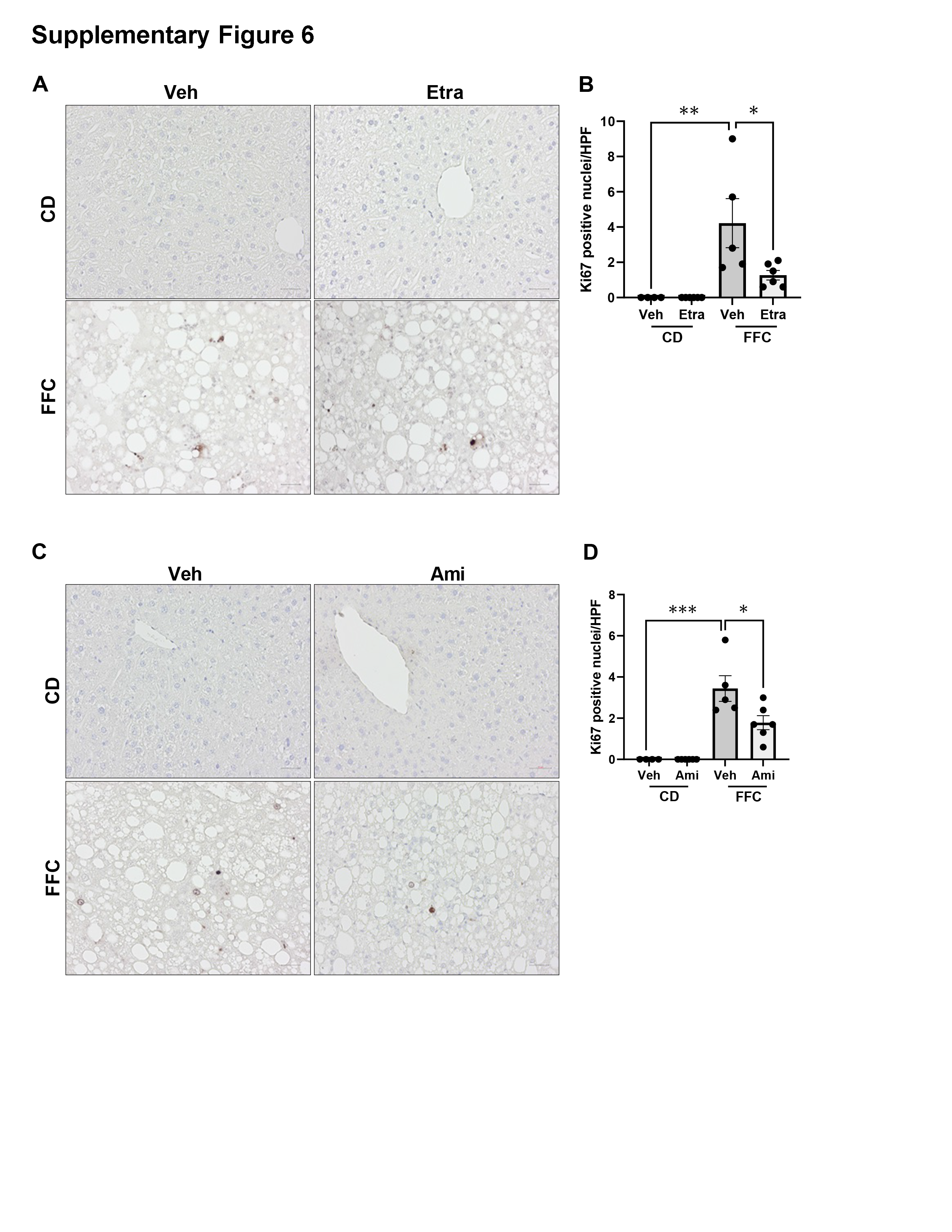

Supplement: Supplementary Figure 6 — FFC diet-induced increase in hepatocyte proliferation is reduced in Etrasimod and Amiselimod treated mice. (A) Representative images showing immunohistochemistry for Ki67 in liver tissue sections for vehicle and Etrasimod treated mice in both CD and FFC cohorts. (B) Positive immunoreactive staining (dark brown) was quantified as Ki67 positive nuclei per high power field in CD and FFC-fed mice treated with vehicle or Etrasimod. CD (vehicle n=4, Amiselimod n=6) and FFC (vehicle n= 5, Amiselimod n=6). (C) Representative images showing immunohistochemistry for Ki67 in liver tissue sections for vehicle and Amiselimod treated mice in both CD and FFC cohorts. (D) Positive immunoreactive staining (dark brown) was quantified as Ki67 positive nuclei per high power field in CD and FFC-fed mice treated with vehicle or Amiselimod. Scale bars represent 50 μm. CD (vehicle n=4, Amiselimod n=6) and FFC (vehicle n= 5, Amiselimod n=6). *p<0.05, **p<0.01, ***p<0.001. [file Image_6.tiff]

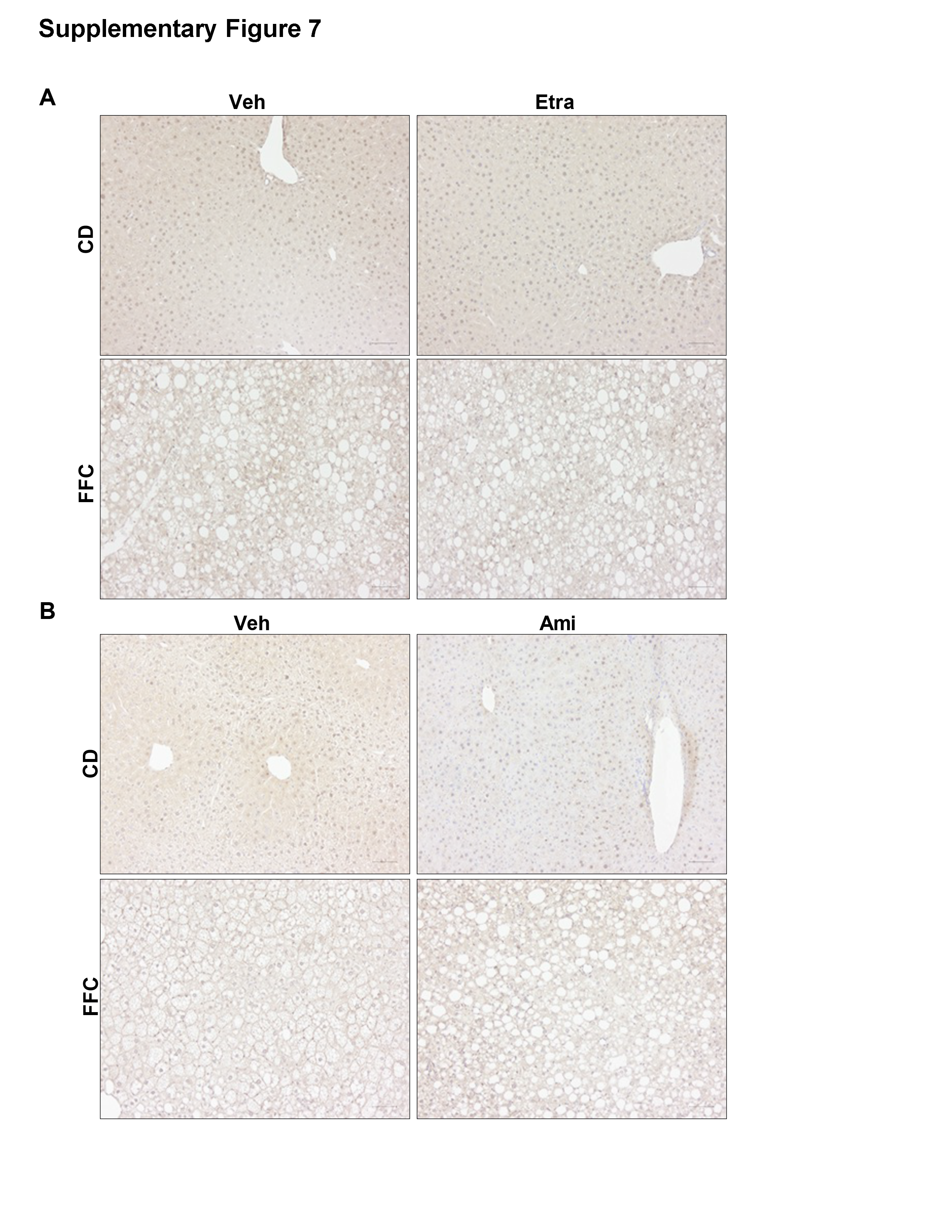

Supplement: Supplementary Figure 7 — Hepatocyte differentiation is comparable in vehicle treated and Etrasimod or Amiselimod treated FFC-fed mouse livers. (A) Representative images showing immunohistochemistry for HNF4α in liver tissue sections for vehicle and Etrasimod treated mice in both CD and FFC cohorts. (B) Representative images showing immunohistochemistry for HNF4α in liver tissue sections for vehicle and Amiselimod treated mice in both CD and FFC cohorts. Scale bars represent 50 μm. [file Image_7.tiff]
